# Thermally induced alloying processes in a bimetallic system at the nanoscale: AgAu sub-5 nm core–shell particles studied at atomic resolution†
†Electronic supplementary information (ESI) available. See DOI: 10.1039/C7NR07286D


**DOI:** 10.1039/c7nr07286d

**Published:** 2018-01-02

**Authors:** Maximilian Lasserus, Martin Schnedlitz, Daniel Knez, Roman Messner, Alexander Schiffmann, Florian Lackner, Andreas W. Hauser, Ferdinand Hofer, Wolfgang E. Ernst

**Affiliations:** a Institute of Experimental Physics , Graz University of Technology , Petersgasse 16 , A-8010 Graz , Austria . Email: andreas.w.hauser@gmail.com ; Email: wolfgang.ernst@tugraz.at ; Fax: +43 (316) 873-108140 ; Tel: +43 (316) 873-8157 ; Tel: +43 (316) 873-8140; b Institute for Electron Microscopy and Nanoanalysis & Graz Centre for Electron Microscopy , Graz University of Technology , Steyrergasse 17 , A-8010 Graz , Austria

## Abstract

Alloying processes in nanometre-sized Ag@Au and Au@Ag core@shell particles with average radii of 2 nm are studied *via* high resolution Transmission Electron Microscopy (TEM) imaging on *in situ* heatable carbon substrates. The bimetallic clusters are synthesized in small droplets of superfluid helium under fully inert conditions. After deposition, they are monitored during a heating cycle to 600 K and subsequent cooling. The core–shell structure, a sharply defined feature of the TEM High-Angle Annular Dark-Field images taken at room temperature, begins to blur with increasing temperature and transforms into a fully mixed alloy around 573 K. This transition is studied at atomic resolution, giving insights into the alloying process with unprecedented precision. A new image-processing method is presented, which allows a measurement of the temperature-dependent diffusion constant at the nanoscale. The first quantification of this property for a bimetallic structure <5 nm sheds light on the thermodynamics of finite systems and provides new input for current theoretical models derived from bulk data.

## Abstract 2

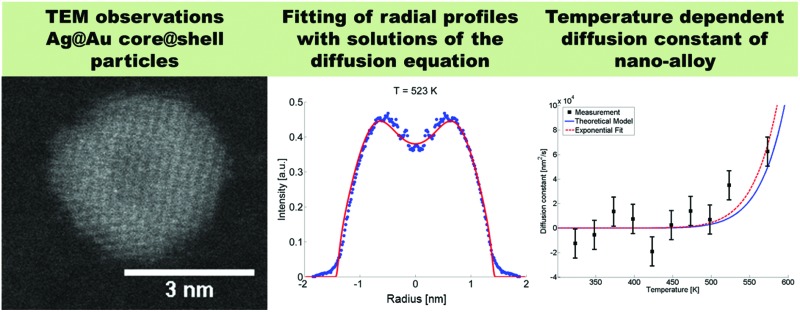
Alloying processes in nanometre-size Ag@Au and Au@Ag core@shell particles are studied *via* high resolution Transmission Electron Microscopy (TEM) imaging.

## 1. Introduction

Core@shell nanoparticles represent a class of materials with unique physical properties and various fine-tuning possibilities with respect to size, morphology and the variety of composition. Due to this extreme flexibility, a wide range of potential applications has been suggested for these materials.^1^–^3^

Among the elements used for nanoparticle synthesis noble metals play a major role.^4^–^6^ The increase in occupation number of the d-orbital and the rise of cohesive energy for elements with higher atomic charge *Z* inside a group in the periodic table, determine silver and gold as the noblest metals within the transition metals.^7^ At the nanoscale the ratio of surface to volume increases dramatically. A higher fraction of atoms at the edges and corners effectively reduces the mean coordination number and is therefore boosting the chemical reactivity.^8^ Under the influence of an electric field, the electrons of these nanostructures show coherent collective oscillations known as plasmon resonances. The combination of gold and silver has highly desirable functionality with possible applications in catalysis and medicine.^9^,^10^ Furthermore, both elements are highly resistant to oxidation under ambient conditions as well as during TEM measurements, and their phase diagram does not show a miscibility gap. Their inertness completely removes any oxidation or contamination issues during the synthesis of the core@shell structure. This set of properties makes the AgAu system ideal for a detailed study of alloying at the nanoscale.

Theoretical approaches such as the CALPHAD (Calculation of Phase Diagrams) have been developed in the past to describe the alloying process in nanosystems.^11^,^12^ These calculations rely entirely on assumptions based on thermodynamic data of the bulk.^13^ However, in the sub 5 nm regime, the surface to volume ratio dramatically increases and quantum effects become important. Thus, nanoscopic thermodynamic properties deviate significantly from their macroscopic counterparts. Related phenomena such as diffusion and alloying are completely unexplored in this size regime. Only for larger AgAu clusters, with radii of several tens of nm, attempts have been made to measure the diffusion *via* laser-induced heating.^14^,^15^

In this article, we introduce a new approach in order to describe the alloying of nanometre-sized bimetallic particles based on thermodynamic data obtained in TEM studies, with unprecedented atomic resolution maintained during the entire heating process. The bimetallic particles are grown under fully inert conditions, and deposited on amorphous carbon substrates for TEM imaging during controlled heating. We show how the diffusion constant can be extracted from TEM images and used to quantify the alloying progress as a function of temperature and time.

For the nanoparticle synthesis we exploit superfluid He nanodroplets (He_N_) as nanolabs for the production of mixed-metallic structures in the nanometre range. The dotation of He_N_ with particles is well established in spectroscopy,^16^–^18^ and has recently been adapted for the controlled production and structure preserving soft deposition of metal clusters.^19^–^28^

Within this method, a beam of He nanodroplets, created *via* a supersonic, adiabatic expansion of pressurized helium through a cooled nozzle, collects metal atoms from vapor when passing a series of pickup zones. Due to the extremely low droplet temperature of 0.37 K (ref. 29) the metal atoms start to coagulate and form clusters inside the droplets, which act as cryostats and fully inert synthesis chambers at the same time. This offers not only the possibility of a sequential doping with different metals in any order but also allows us to study metastable structures which are not accessible with conventional techniques. The low temperature of the droplets and their ability to effectively dissipate the binding energies released during the formation of metallic bonds enables the synthesis of cluster geometries far from the global thermodynamic minimum. Another large advantage is the ability to grow clusters without any solvent- or template-induced effects. Also, typical problems of the solution-based synthesis such as large differences in the reduction potentials and a reduced miscibility at lower temperatures^30^,^31^ are avoided.

Our article is structured as follows. Details of the experimental setup are presented in section 2. In section 3, we provide the theoretical background for the proposed relation between two-dimensional scans over TEM image intensities and the diffusion constant. This quantity is then used to evaluate the alloying behaviour for the investigated systems as a function of the temperature. Section 4 is dedicated to an overall comparison of the observed diffusion processes in the AgAu system to predictions of Lee *et al.*,^32^ who suggested a method to derive particle-size dependent phase diagrams from known values of the bulk.

## 2. Experimental setup

For details of the experimental setup we refer to ref. 21. In short, He gas with a purity of 99.9999% and a constant pressure of 20 bar is expanded through a 5 μm nozzle, which is kept at temperatures below 8 K, into a vacuum of ∼10^–5^ mbar. Depending on the nozzle temperature different mean droplet sizes can be produced.^16^ After croping with a 400 μm skimmer, the resulting He beam passes the pickup chamber (base pressures of ∼10^–7^ mbar). Here, the helium droplets are doped with the desired metals while crossing through two separate pickup cells. Inside these cells, the desired metal species are heated to generate enough vapour pressure for pickup. The probability for particle pickup, the mixing ratio as well as the total amount of metal in each droplet can be controlled *via* separate adjustments of the individual cell temperature. Metal atoms captured by the helium droplets start to agglomerate and form cluster or wire-like structures during the flight through the vacuum chamber. Wire-like structures are created if the superfluid helium droplets exhibit quantum vortices,^33^,^34^ a phenomenon which has been observed in the size regime beyond 3 × 10^8^ helium atoms per droplet.^23^ The energy which is released during metal bond formation causes a partial evaporation of helium atoms from the droplets. This is monitored by a quadrupole mass spectrometer. After pickup, the helium beam is again collimated with a 2 mm skimmer and enters the measurement chamber with a base pressure of ∼5 × 10^–10^ mbar. The bimetallic clusters are deposited onto heatable TEM grids (DENSsolutions Nano-Chip XT carbon) in a soft-landing process where any remaining He is vaporized.^22^,^26^,^35^

### 2.1. Nanoparticle synthesis

The cold He environment allows for a controlled synthesis of core@shell structures in any desired ordering *via* sequential doping. Depending on the original He droplet size before the pickup of metal atoms and the vapour pressure in the pickup cells it is possible to create either core@shell cluster particles or core@shell nanowires. Details of the latter procedure can be found in ref. 36 and 37, where the phenomenon of quantum vortices was exploited to obtain enhanced one-dimensional growth. First observed in bulk superfluid helium (He II),^38^–^40^ vortices can attract dopants which are immersed in the helium due to a pressure gradient around the vortex core,^41^,^42^ which causes a preference for wire-like structures.^43^–^45^

In the current manuscript, we focus on the regime of core@shell nanoparticles produced at a nozzle temperature of 6.7 K and a pressure of 20 bar. This corresponds to an average size of *N* ∼ 10^7^ helium atoms per helium droplet.^16^ At these conditions, metal clusters with diameters ≤5 nm are produced inside the He droplets. Their shape is best described as spherical.^27^ For the synthesis of nanometre-sized core@shell clusters we have chosen a helium droplet dotation ratio of 20 at% core and 80 at% shell material. On average, clusters with a radius of 2 nm are produced, which corresponds to approximately 2500 metal atoms per cluster.

### 2.2. Data acquisition

After deposition on a heatable amorphous carbon TEM grid the particles are studied *via* High-Angular Annular Dark-Field (HAADF) imaging using a FEI Titan^3^ G2 60-300. A Gatan quantum energy filter attached to the microscope is employed for Electron Energy Loss Spectroscopy (EELS). Complementary, a four-quadrant Energy-Dispersive X-ray spectroscopy (EDX) detector (FEI Super-X) is utilized. The temperature of the heatable carbon grid can be varied between room temperature and 1500 K with heating rates of up to 200 K ms^–1^. In the presented experiments the temperature is increased from room temperature to 573 K in steps of 25 K.

## 3. Results

**Fig. 1:**
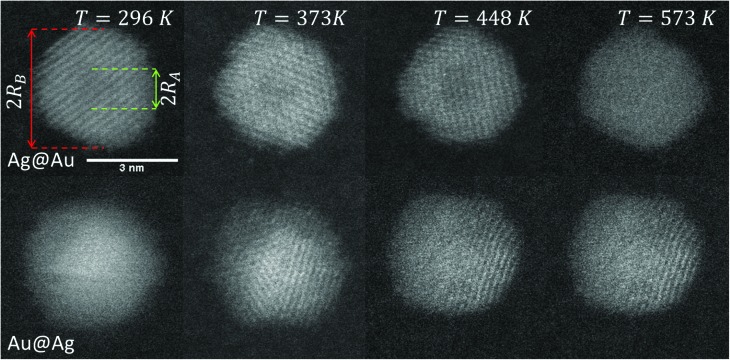
Transmission electron microscopy HAADF scans of a single Ag@Au core@shell cluster as a function of temperature (upper images) and a Au@Ag core@shell cluster scanned at the same temperatures (lower images). With increasing temperature, a softening of the contrast borders between Ag and Au is detected.

Our approach enables us to perform *in situ* observations of the nanoscale alloying of Au and Ag core@shell nanoparticles with atomic site resolution. Fig. 1 shows an example of a Ag@Au (top panel) and Au@Ag (bottom panel) core@shell nanoparticle at different substrate temperatures. With the recorded HAADF intensity being proportional to *Z*^2^ (with *Z* as the atomic number of the corresponding element),^46^ it is easily possible to distinguish between the bright gold atoms and the darker silver atoms. With increasing temperature, the initially separated elements diffuse into each other. In particular, when comparing images taken at 448 K to those taken at 573 K, an intact core can still be seen at the former temperature but disappears completely at the latter.

### 3.1. From density profiles to a diffusion constant *D*(*T*)

We present a convenient technique for the extraction of the temperature-dependent diffusion constant of a given metal combination directly from HAADF observations. As described in section 2.1, our synthesis method allows the full encapsulation of any metallic core material A by a metal of type B or *vice versa*. Assuming a spherically symmetric cluster, the initial, radial density profiles *ρ*_A_(*r*, *t* = 0) and *ρ*_B_(*r*, *t* = 0) of the core and the shell, respectively, can be approximated by an analytical expression *d*(*r*, *R*) built from error functions,1
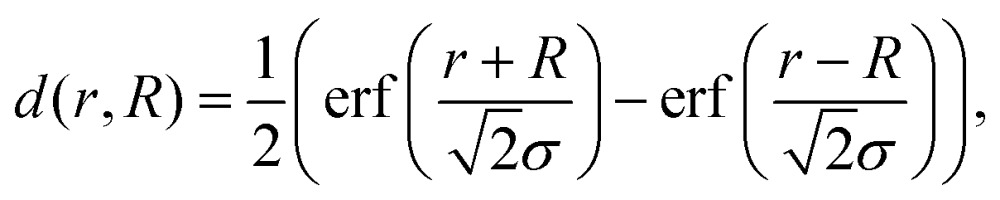
with the definitions

**Fig. 2:**
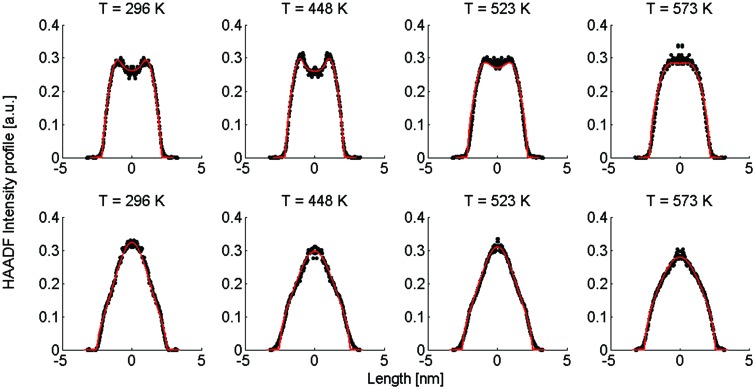
Linear HAADF intensity profiles of a Ag@Au (upper row) and Au@Ag (lower row) cluster as a function of the observation temperature. For each temperature a fit of the calculated intensity profile and the measurements is performed. Each measured, temperature-dependent intensity profile is plotted and compared to the calculated fit obtained from eqn (4).

2 *ρ*_A_(*r*, 0) = *d*(*r*, *R*_A_),

3 *ρ*_B_(*r*, 0) = *d*(*r*, *R*_B_) – *d*(*r*, *R*_A_),

with *R*_A_ denoting the core radius and *R*_B_ the outer radius of the bimetallic cluster, as indicated in the upper left image of Fig. 1. The slope of the density profile is described by the parameter *σ* which controls the smoothness of the density progression at the transition zone from metal A to B. These initial profiles *ρ*_*i*_(*r*, *t*) (for *i* = A, B) are then numerically evolved in time as described by the spherical Einstein diffusion equation,^47^4
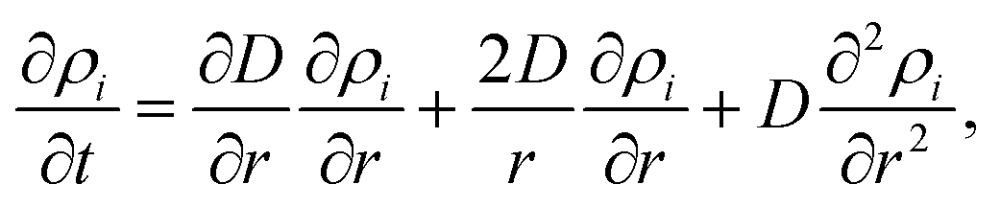
with the diffusion constant *D* entering the equation on the right hand side. Note that our model considers metal diffusion processes of a finite spherical object, which is reflected in the choice of setting *D* = 0 for *r* > *R*_B_. With this assumption and the initial condition *ρ*_*i*_(*r*, 0) = *ρ*_*i*,0_eqn (4) can be solved numerically *via* a finite differences approach. The choice of Gauss error functions for the initial shell density keeps the derivative of the first term on the right hand side of eqn (4) finite. Note that this term vanishes in the standard form of the diffusion equation for a constant *D*; its presence here is a consequence of the finite cluster size and prevents the system from an unphysical diffluence. The solution *ρ*_*i*_(*r*, *t*) is then used to obtain a simulated intensity profile of the cluster as a function of the diffusion progress. For a direct comparison to the two-dimensional, experimentally accessible observable, the radial density distribution has to be converted into an intensity profile. With the intensity being proportional to the projected density, such a profile is obtained by the integration of 
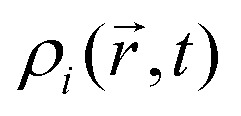
, the corresponding spatial density distribution, over one cartesian coordinate, and plotting the result as a function of one of the remaining coordinates. A comparison of the simulated profiles to the angularly averaged TEM data for Ag@Au and Au@Ag clusters can be found in Fig. 2.

Note that this figure already contains a series of comparisons for different temperatures, which is, to the knowledge of the authors, the first detailed documentation of a diffusion process over temperature in bimetallic nanoparticles with atomic resolution. It can be seen that the border between the core and shell element flattens out with increasing temperature. For Ag@Au clusters (upper panel), the initial form has a local intensity minimum in the centre of the cluster and a maximum at the radius of the core. This is due to the projection of a sphere when performing two-dimensional scans, with the intensity profile being obtained by the integration over elements in *z*-direction. In the case of Au@Ag, the intensity profile shows a global maximum in the centre, where the amount of projected Au atoms is at a maximum, and an inflection point at the core radius. The shape of the profile for the Ag@Au cluster changes significantly with temperature, but only slightly for the Au@Ag cluster, which results in larger uncertainties for the diffusion progress in the latter case. Therefore, only Ag@Au clusters are used to determine the temperature-dependent diffusion constant *D*(*T*) for the Ag@Au system in the next step.

First, note that the temperature dependence enters eqn (4) only *via* the diffusion constant *D* = *f*(*T*). Neglecting the first term on the right hand side of eqn (4), a mathematical necessity to keep the system size finite over time, it becomes obvious that the solution of the differential equation can be written as *ρ*_*i*_(*r*, *D*(*T*)·*t*), a function of the radius and the product of the time-dependent diffusion constant and the time. Therefore, information for *D* can be derived by solving eqn (4) within a given time interval *k* between two TEM-profile measurements: since the temperature is increased in discrete steps and kept constant between two measurements, the diffusion constant of each interval *k* is determined by the current temperature *T*_*k*_ and by the change of the density profiles between the beginning and the end of the interval. The diffusion constant for interval *k* is obtained *via* least-square fits of the time-evolved profiles to the measured, angular-averaged TEM results. From these point-wise evaluations we can determine the approximate temperature dependence of *D* within the experimentally accessible heating range.

### 3.2. *D*(*T*) for finite systems

**Fig. 3:**
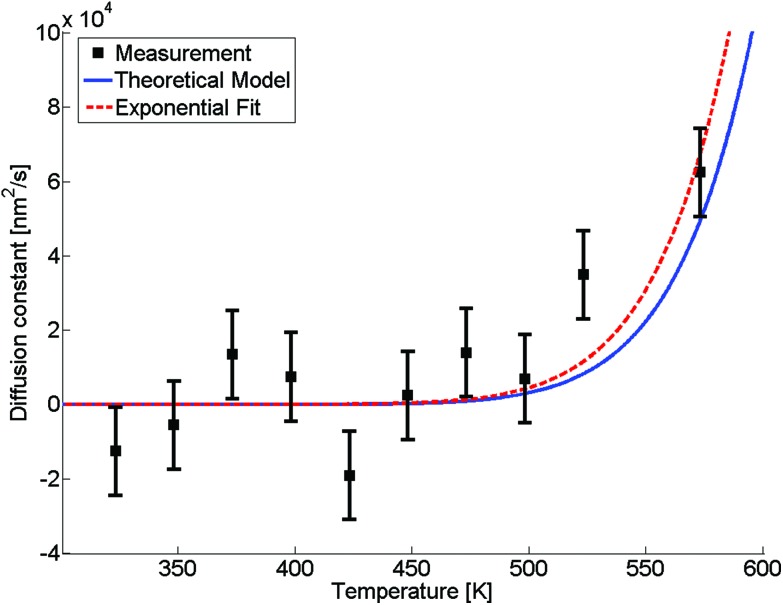
Diffusion constant as a function of temperature. Black squares are derived from HAADF measurements, the red dashed line is a fit based on eqn (5), the blue line is based on a revised model from ref. 49 derived from bulk values with a mean radius of the cluster of *r* = 1.95 nm and atomic binding length of *h* = 0.2889 nm.

The evaluated dependence of the diffusion constant on the temperature is plotted in Fig. 3 as determined from the HAADF images *via* the method discussed above. The uncertainty in these measured points is dominated by fluctuations in image contrast due to minimal changes in the electron current. Other factors such as the limited resolution and deviations of the clusters from the assumed spherical shape are comparably small. An exponential fit of the data (red dashed line) has been added to the graph, inspired by the temperature dependence which is typically observed for bulk,

5 *D*(*T*) = *D*_0_ exp(–*α*/*T*),

with a literature value of *D*_0_ = 7.2 × 10^–6^ m^2^ s^–1^ for silver in gold^48^ and *α* as the only fitting parameter. This approximation of the diffusion constant, which is, according to ref. 48, also depending on the actual Ag/Au mixing ratio, is justified by the small size of the silver core, where a large fraction of Ag atoms is sitting directly at the contact surface to the pure Au shell. We obtain a value of *α* = 7.325 × 10^5^ K^–1^. The fitted curve suggests the occurrence of fully mixed nanoalloys at temperatures of approximately 500 K and above. We further compare the results of our TEM-based ansatz to a theoretical model based on the extrapolation from well-known bulk parameters.^49^

Giving a brief outline of this approach to approximate the size-dependent diffusion constant *D*(*r*), we start from eqn (5) to obtain the temperature- and size-dependent diffusion constant by writing *α* as –*E*(*r*)/*R*, with *E*(*r*) as the thermal activation energy and *R* as the ideal gas constant. The thermal activation energy can be related to the melting temperature *T*_m_(*∞*) of the bulk *via* a constant *C*,

6 *E*(*∞*) = *CT*_m_(*∞*).

Note that the constant *C* depends on the material and on the type of diffusion, but not on the particle size. We further assume that any size dependence of the activation energy only enters *via* a dependence of the melting temperature on *r*,^50^

7 *E*(*r*) = *CT*_m_(*r*),

with *r* denoting the radius of the spherical cluster. Combining eqn (6) and (7), the thermal activation energy *E*(*r*) for a finite system can be expressed as8
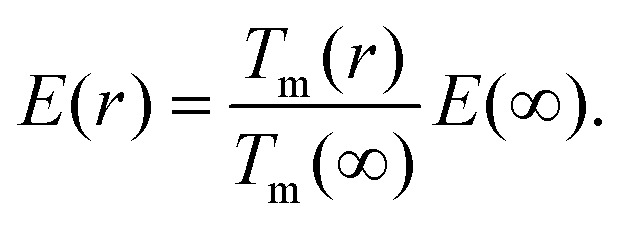



The size-dependent diffusion coefficient can be rewritten as9
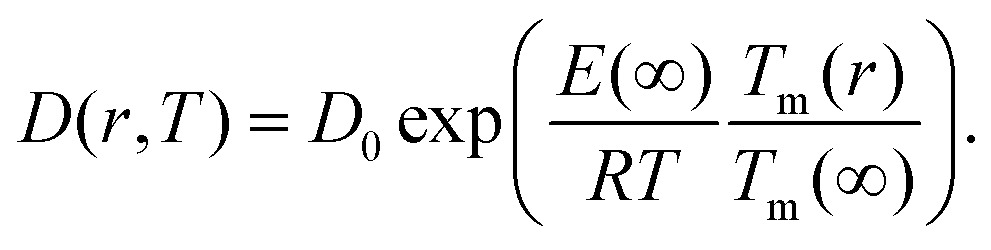



Several experimental and theoretical studies have shown that the activation energy decreases with decreasing size.^51^,^52^ In an attempt to obtain a direct functional dependence of *D* on the particle size *r*, we employ Lindemann's empirical melting criteria to rewrite the factor *T*_m_(*r*)/*T*_m_(*∞*) in eqn (9) by a geometrically motivated expression. In this crude approximation, melting is assumed to take place if the average amplitude of thermal vibrations exceeds a critical value.^53^ For the given case, the following expression can be deduced,^54^10
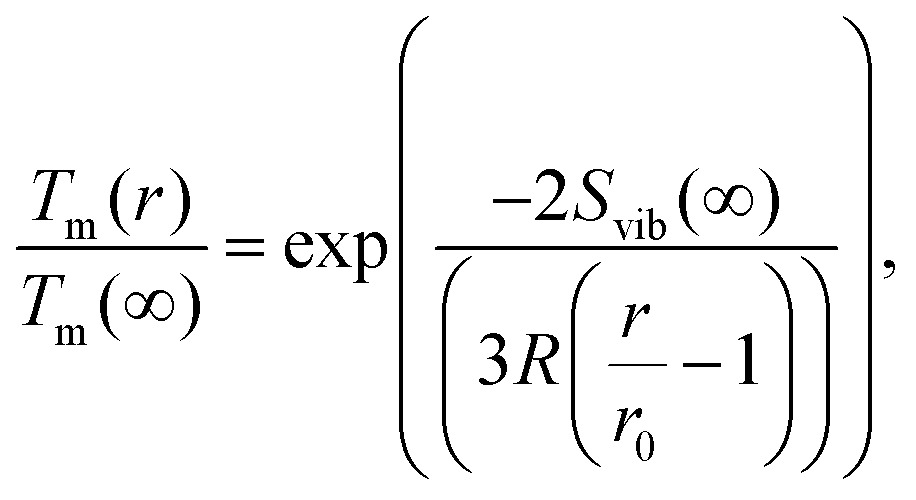
with *S*_vib_ as the melting entropy of the bulk and *r*_0_ as a geometry dependent factor defined as *r*_0_ = *h*(3 – *d*), with *d* = 0 for spherical clusters and *h* = 0.2889 nm as the atomic diameter. Combining eqn (5), (9) and (10), the diffusion constant can be written as11
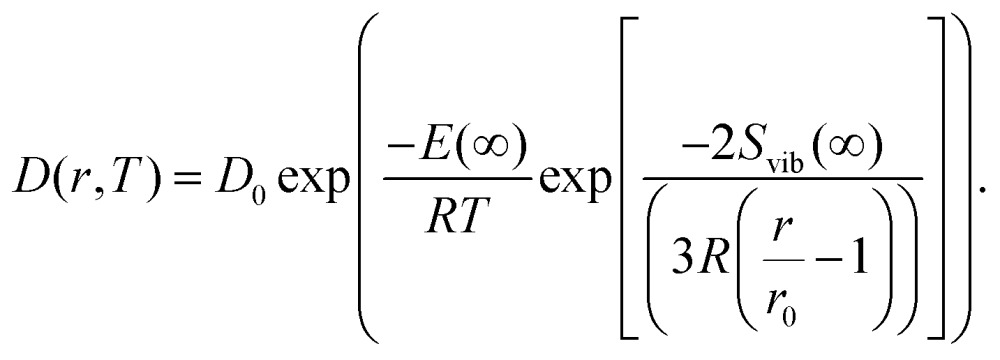



The size-dependence of all relevant parameters in eqn (11) can be extracted from the literature: the melting entropy *S*_vib_ of nanoparticles with *r* = 2 nm increases approximately by 9% in comparison to the bulk,^55^ for which a reference value of *S*_vib_ = 9.157 J mol^–1^ K^–1^ is used.^49^ The size dependence of *D*_0_, on the other hand, can be fully neglected.^52^ We use a value of *D*_0_ = 7.2 × 10^–6^ m^2^ s^–1^ (Ag in Au).^48^ The activation energy of the bulk is assumed to have a value of *E*(∞) = 169 800 J mol^–1^.^49^ The resulting curve is shown as blue line in Fig. 3. Note that our ansatz is indeed capable to estimate the onset temperature for full diffusion as it is observed in the experiment. Furthermore, the slope of the curve deviates only minimally from the exponential fit (red dashed line) of the data points. Note that the model is purely based on literature values and physically motivated extrapolations thereof. The excellent match of experiment and model supports our claim to have found a useful, novel experimental technique for the exploration of metal diffusion and mixing at the nanoscale. This is nicely illustrated by a direct comparison of the activation energy *E*(*r*) for the given particle size *r* = 1.95 nm derived from the fit (–8.9573 × 10^4^ J mol^–1^) to the value obtained from the model (–8.8098 × 10^4^ J mol^–1^), which deviates from the former by less than 2 percent.

## 4. Phase diagrams at the nanoscale

**Fig. 4:**
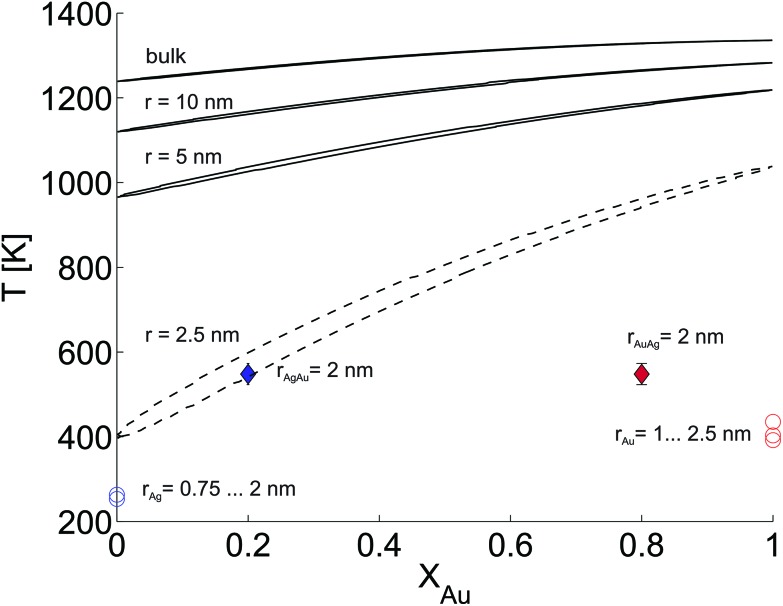
Phase diagram for the AgAu system as a function of the particle radius as suggested in ref. 32, compared to selected measurements of crucial temperatures in pure and mixed-metallic systems, see text for details.

In this last section we look at our experimental results in the light of ongoing discussions about the impact of particle size on fundamental qualities of bimetallic phase diagrams. The correct description and even the definition of phase transitions becomes highly problematic at the nanoscale. This is indicated in Fig. 4, which contains a direct comparison of selected recent measurements of our group on pure Ag and Au nanostructures,^28^ the mixed-metallic AgAu cluster data of the current study, and a reference phase diagram based on a numerical model describing the impact of the particle radius on the solidus and liquidus curves of the AgAu phase diagram (see the ESI† for details).^32^

The graph documents the dependence of the liquid-to-solid transition on the temperature *T* and the relative content *X*_Au_ of gold in the binary mixture. The AgAu system forms a binary solution with unlimited solubility in both phases and shows the typical lenticular shape for the two-phase region. However, this area of phase coexistence is barely visible at the chosen temperature scale in the figure, although its average width increases slightly with decreasing particle radius.

For particles with a radius of 2.5 nm the onset of melting is predicted to occur at 400 K for pure Ag clusters and at 1000 K for pure Au clusters, with a monotonic increase of the melting temperature with increasing amount of gold. This theoretical prediction can be related to measured quantities as follows. In the case of the pure metal structures, the onset temperature of Rayleigh breakup, a well-understood process of diffusion on a metal surface driven by the minimisation of surface energy,^25^,^28^,^56^ can be interpreted as a lower limit for the solidus curve since breakup becomes visible before the total melting of the structures upon heating. These results are plotted as circular data points at *X*_Au_ = 0 and *X*_Au_ = 1.^57^ In the case of the mixed-metallic particles we refer to Fig. 1 presented above, which clearly shows an ordered structure even for a temperature of 573 K where complete intermixing is observed. The corresponding data points are plotted as diamonds in Fig. 4. These findings confirm two things: first, as stated above, alloying at the nanoscale is a temperature-driven diffusion process and clearly distinguishable from a first-order phase transition such as melting. Second, in comparison to predictions for the phase diagram in this size regime, our experimental data suggests a concave form of the solidus-liquidus curve, *i.e.* an increased temperature-stability of bimetallic, alloyed particles with melting temperatures which lie above that of the more stable element.

This unexpected property of the phase diagram at the nanoscale can not be derived from bulk data *via* the suggested scaling of model parameters with respect to particle diameter. We note that the authors of ref. 32 give a lower limit of 5 nm for the applicability of their numerical model, but it can be extrapolated down to radii of about 2.5 nm before getting ill defined. In this size regime, a large fraction of atoms is located at the surface or at interfaces, and it is no longer possible to derive the correct behaviour from bulk data as the surface diffusion becomes the main driving mechanism for the alloying process. As a result, the 1/*r* extension of certain parameters in the numerical model, a core feature of this ansatz, is no longer justified and needs readjustment.

However, besides showing that current models need to be revised before application to the studied size regime, our experimental findings also indicate new opportunities for the design of temperature-stable structures at the nanoscale.

## 5. Conclusion

In this article we reported on *in situ* nanoscale diffusion of core@shell gold and silver clusters under subsequential heating. The nature of the alloying process was studied *via* atomic resolution TEM imaging.

Using HAADF images we developed a method for the determination of the diffusion constant *D* as a function of temperature. This technique is generally applicable to any metal combination with sufficiently large differences in the atomic number of the elements involved, as HAADF contrast is proportional to *Z*^2^. The method is based on a time evolution of the actual particle density according to Einstein's diffusion equation, and correlates the density at each step with its corresponding TEM intensity profile along radial scans of the metal particles.

We observe that the diffusion on this length scale (≤5 nm) is initiated at lower temperatures than in the corresponding bulk material, which is a consequence of dominant surface size effects in this regime. For both systems (Ag@Au and Au@Ag), alloying takes place between 500 and 550 K.

The results of our novel TEM-based ansatz for the derivation of *D*(*T*) are compared to a theoretical model based on extrapolation of bulk parameters. We find an excellent agreement between theory and experiment in terms of absolute values as well as overall functional dependence on *T*, which renders our experimental technique a useful tool for future studies of metal diffusion and alloying processes at the nanoscale. We note that the nanoparticles remain solid upon complete mixing. This shows that alloying at this size regime is a temperature-driven diffusion process and clearly distinguishable from a first-order phase transition.

A final comparison of experimental data to theoretical phase diagrams for the AgAu system at the nanoscale suggests a revision of current models. The experiment indicates a concave form of the solidus-liquidus curve, or, in other words, an increased temperature-stability of bimetallic, alloyed particles with melting temperatures above those of the pure elements at the given cluster size. This novel, unexpected feature can not be derived from known models based on a size-dependent rescaling of bulk parameters.

## Conflicts of interest

There are no conflicts to declare.

## Supplementary Material

Supplementary information
