# Supplementary material for: Thermally induced alloying processes in a bimetallic system at the nanoscale: AgAu sub-5 nm core–shell particles studied at atomic resolution
Source: Nanoscale. 2018 Jan 2;10(4):2017–24. doi: 10.1039/c7nr07286d (PMC5795486; doi:10.1039/c7nr07286d)
Supplement: Supplementary file 1 [file NR-010-C7NR07286D-s001.pdf]

Thermally induced alloying processes in a bimetallic system at the nanoscale: AgAu sub-5 nm core-shell particles studied at atomic resolution

Maximilian Lasserus,[†] Martin Schnedlitz,[†] Daniel Knez,[‡] Roman Messner,[†] Alexander Schiffmann,[†] Florian Lackner,[†] Andreas W. Hauser,[*,†] Ferdinand Hofer,[‡] and Wolfgang E. Ernst[*,†]

†*Institute of Experimental Physics, Graz University of Technology, Petersgasse 16, A-8010 Graz, Austria. Fax: +43 (316) 873-108140; Tel: +43 (316) 873-8157; +43 (316) 873-8140;*
‡*Institute for Electron Microscopy and Nanoanalysis & Graz Centre for Electron Microscopy, Graz University of Technology, Steyrergasse 17, A-8010 Graz, Austria.*

E-mail: andreas.w.hauser@gmail.com; wolfgang.ernst@tugraz.at

In the first section of this Supporting Information we present a schematic of our experimental setup. In Section 2 additional TEM images of bimetallic core@shell clusters is provided. The application of the CALPHAD method to the AgAu system is demonstrated in Section 3. This section contains all formulas and parameters needed to generate phase diagrams for bimetallic AgAu particles of any radius and composition.

1 Details of the Experimental Apparatus

In order to provide a more detailed description of the actual experimental setup we present a schematic of the whole apparatus in Figure 1. The setup consists of three vacuum chambers, one for the helium droplet production (Source Chamber SC), one for the dotation of the droplets with metal species (Pickup Chamber PC) and one for cluster analysis and deposition on TEM grids (Main Chamber MC).

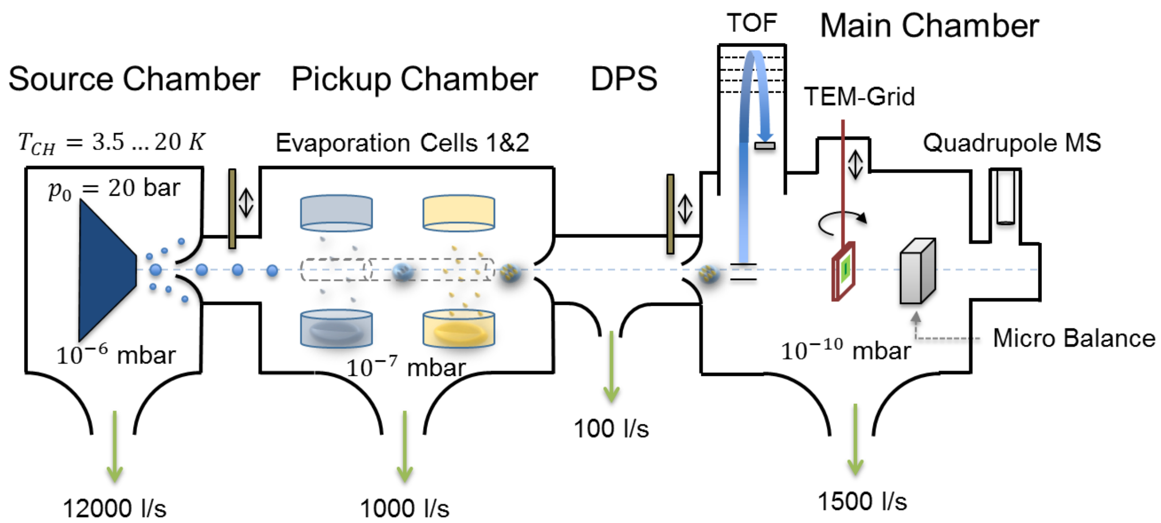

Figure 1: Schematic of the experimental setup used for the synthesis of metal clusters inside liquid He droplets; see text for details.

In the SC, a beam of liquid He droplets is produced by the expansion of He at 20 bar into vacuum through a nozzle cooled by a cold head with temperatures between 3.5 and 20 K. The droplets are then doped inside the PC with up to two metal species. The metal atoms stick to the droplets and form clusters inside during their time of flight from the PC through a differential pumping stage (DPS) to the MC. Inside the MC, a TOF-MS is used for the *in situ* analysis of the cluster size either by electron impact ionisation or via laser-induced photoionisation. A quartz crystal microbalance is used for the determination of the total amount of deposited metal. A quadrupole mass spectrometer is used to analyse the remaining residual gas. It allows us to determine the total amount of helium flow as well as the actual size of He droplets. The MC also contains a manipulator for the insertion and

the adjustment of TEM grids or other supports.

The size of the metal clusters can be adjusted via the size of the helium droplets which is manipulated via the stagnation temperature, the pressure, and the temperature of each evaporation cell in the PC. Before depositing clusters on heatable TEM grids, their size distribution is analysed via a time-of-flight mass spectrometer, the micro-balance, and the monitoring of the evaporated helium via a quadrupole mass spectrometer.

2 Additional TEM imagery

In this section additional HAADF TEM studies of core@shell clusters are provided. The experimental studies have been performed with a FEI Titan^3 G2 60-300 at 300 kV. In Figure 2, the images of nine different core@shell clusters at room temperature are presented. Pictures 1-5 show Ag@Au configurations, pictures 6-9 depict Au@Ag configurations. As mentioned in the main article, the difference in the intensity is caused by the different Z-contrast of gold and silver, allowing for a visual discrimination of core and shell areas with this technique.

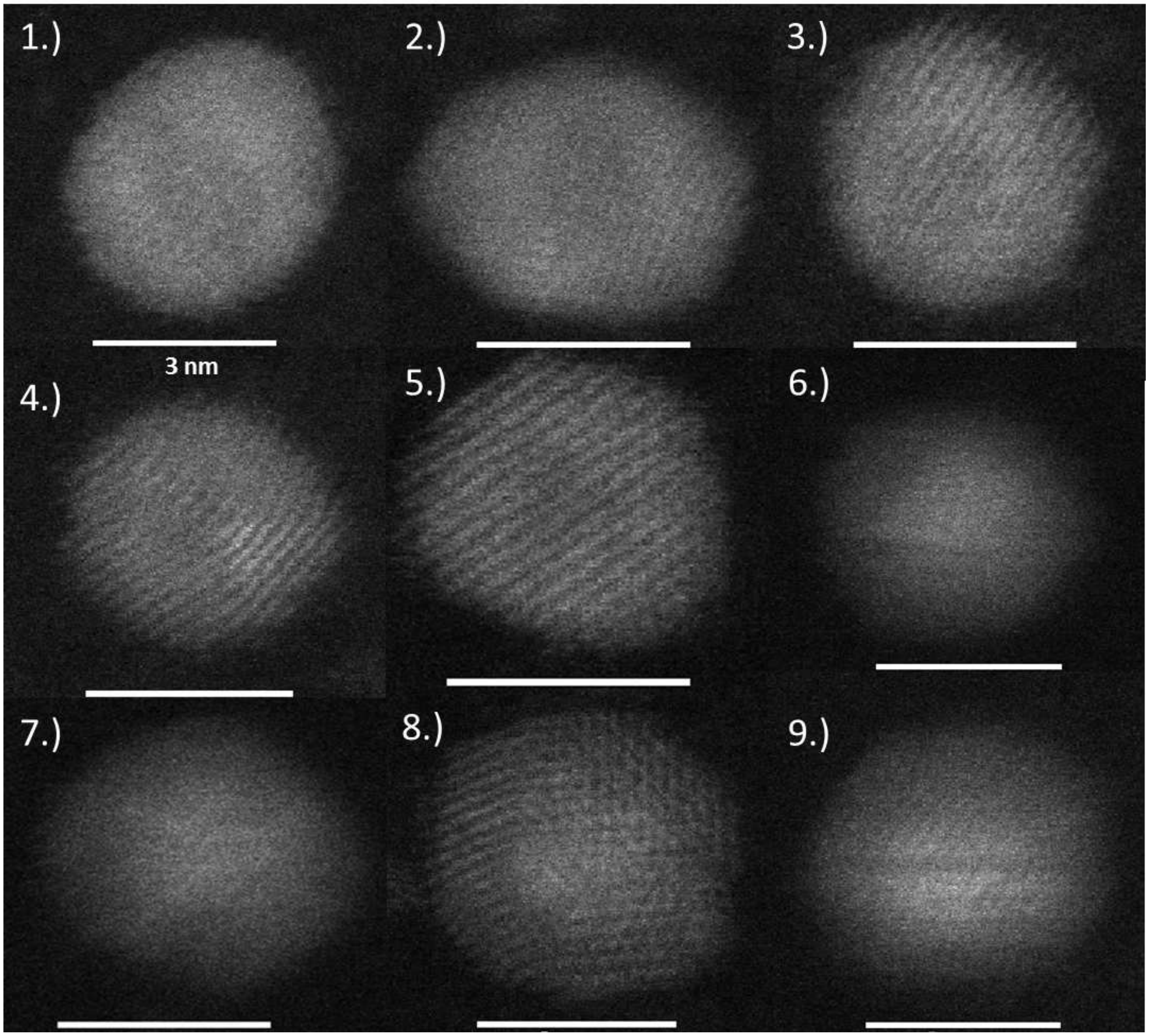

Figure 2: TEM images of five Ag@Au clusters (1-5) to four Au@Ag clusters (6-9), taken at room temperature. The inverted structure is obvious from the difference in contrast, although the core-shell feature is optically more pronounced if silver is the core element encapsulated in a shell of gold.

3 Application of the CALPHAD method to the AgAu system

In this section we list the reassessed Gibbs energies which are used to calculate the phase diagrams for the AuAg alloy as a function of the particle size and temperature for the liquid (L) and the solid (S) phase. The numerical values are taken from Ref. 1.

$$
\begin{aligned}
G^L_{Ag}(T<1234,r) &= \left(3815.93+\frac{2.462\cdot 10^{-5}}{r}\right)+\left(109.302897-\frac{1.91\cdot 10^{-9}}{r}\right)T \\
&- 23.8463316T\log(T)-\left(0.001790585+\frac{5.18\cdot 10^{-13}}{r}\right)T^2 \\
&- 3.98587\cdot 10^{-7}T^3-12011T^{-1}-1.04452328\cdot 10^{-20}T^7 \\
G^L_{Ag}(T>1234,r) &= \left(-3583.962+\frac{2.46\cdot 10^{-5}}{r}\right)+\left(180.95395-\frac{1.91\cdot 10^{-9}}{r}\right)T \\
&- 33.472T\log(T)+\frac{-5.18\cdot 10^{-13}}{r}T^2 \\
G^L_{Au}(T<1336.15,r) &= \left(-3352+\frac{2.73\cdot 10^{-5}}{r}\right)+\left(215.885-\frac{7.98\cdot 10^{-10}}{r}\right)T \\
&- 40.73T\log(T)+\left(0.0128756-\frac{2.18\cdot 10^{-13}}{r}\right)T^2-2.535266\cdot 10^{-6}T^3 \\
&+ 846536T^{-1}-7.069217\cdot 10^{9}T^{-3}-3.5899325\cdot 10^{-21}T^7 \\
G^L_{Au}(T>1336.15,r) &= \left(23570+\frac{2.73\cdot 10^{-5}}{r}\right)+\left(89.502-\frac{7.98\cdot 10^{-10}}{r}\right)T \\
&- 23.454T\log(T)-\left(2.18\cdot\frac{10^{-13}}{r}\right)T^2-8892561.16T^{-1} \\
G^S_{Ag}(T<1234,r) &= \left(-7209.512+\frac{4.75\cdot 10^{-5}}{r}\right)+\left(118.200734-\frac{1.33\cdot 10^{-8}}{r}\right)T \\
&- 23.8463316T\log(T)-0.001790585T^2-3.98587\cdot 10^{-7}T^3-12011T^{-1} \\
G^S_{Ag}(T>1234,r) &= \left(-15094.864+\frac{4.75\cdot 10^{-5}}{r}\right)+\left(190.26484-\frac{1.33\cdot 10^{-8}}{r}\right)T \\
&- 33.472\log(T)-1.40918321\cdot 10^{29}T^{-9}
\end{aligned}
$$

$$
\begin{aligned}
G^S_{Au}(T<1336.15,r) &= \left(-15745+\frac{4.380\cdot 10^{-5}}{r}\right)+\left(225.142-\frac{9.67\cdot 10^{-9}}{r}\right)T \\
&- 40.73T\log(T)+0.0128756T^2-2.535266\cdot 10^{-6}T^3+846536T^{-1} \\
&- 7.069217\cdot 10^9 T^{-3} \\
G^S_{Au}(T>1336.15,r) &= \left(10886+\frac{4.380\cdot 10^{-5}}{r}\right)+\left(98.987-\frac{9.67\cdot 10^{-9}}{r}\right)T \\
&- 23.454T\log(T)-8892561.1T^{-1}+1.72894275\cdot 10^{29}T^{-9} \\
G^L_{Ex}\left(X_{Au},X_{Ag},T,r\right) &= X_{Au}X_{Ag}\left(\left(-16402\frac{3.22\cdot 10^{-7}}{r}\right)+\left(1.14-\frac{6.834\cdot 10^{-10}}{r}\right)T\right) \\
&+ \left(\frac{-7.987\cdot 10^{-7}}{r}+\frac{3.349\cdot 10^{-10}}{r}T\right)\left(X_{Ag}-X_{Au}\right) \\
&+ \left(\frac{-1.04\cdot 10^{-8}}{r}+\frac{2.373\cdot 10^{-10}}{r}T\right)\left(X_{Ag}-X_{Au}\right)^2 \\
G^S_{Ex}\left(X_{Au},X_{Ag},T,r\right) &= X_{Au}X_{Ag}\left(\left(-15599-\frac{1.6484\cdot 10^{-6}}{r}\right)+\frac{-5.7866\cdot 10^{-10}}{r}T\right) \\
&+ \left(\frac{-1.9979\cdot 10^{-9}}{r}+\frac{-2.8715\cdot 10^{-10}}{r}T\right)\left(X_{Ag}-X_{Au}\right) \\
&+ \left(\frac{-1.30421\cdot 10^{-7}}{r}+\frac{-6.755\cdot 10^{-11}}{r}T\right)\left(X_{Ag}-X_{Au}\right)^2
\end{aligned}
$$

With these expressions, a total Gibbs free energy for both the liquid and the solid phase can be calculated,

$$
\begin{aligned}
G^L_{tot}\left(X_{Au},X_{Ag},T,r\right) &= G^L_{Ex}\left(X_{Au},X_{Ag},T,r\right)+X_{Au}G^L_{Au}(T,r)+X_{Ag}G^L_{Ag}(T,r) \\
&+ RT\left(X_{Au}\log\left(X_{Au}\right)+X_{Au}\log\left(X_{Au}\right)\right), \\
G^S_{tot}\left(X_{Au},X_{Ag},T,r\right) &= G^S_{Ex}\left(X_{Au},X_{Ag},T,r\right)+X_{Au}G^S_{Au}(T,r)+X_{Ag}G^S_{Ag}(T,r) \\
&+ RT\left(X_{Au}\log\left(X_{Au}\right)+X_{Au}\log\left(X_{Au}\right)\right),
\end{aligned}
$$

(1)

which determines the phase (liquid, solid, coexistence) of the system for any metal ratio, particle radius and temperature.

References

(1) Lee, J.; Sim, K. J. General equations of CALPHAD-type thermodynamic description for metallic nanoparticle systems. *Calphad* **2014**, *44*, 129 – 132sol, Special Issue: TOFA 2012 Discussion Meeting on Thermodynamics of Alloys.
